# Evidencing the existence of exciting half-metallicity in two-dimensional TiCl_3_ and VCl_3_ sheets

**DOI:** 10.1038/srep19407

**Published:** 2016-01-18

**Authors:** Yungang Zhou, Haifeng Lu, Xiaotao Zu, Fei Gao

**Affiliations:** 1 School of Physical Electronics, University of Electronic Science and Technology of China, Chengdu, 610054, P.R. China; 2 Institute of Fundamental and Frontier Sciences, University of Electronic Science and Technology of China, Chengdu, 610054, P. R. China; 3 Department of Nuclear Engineering and Radiological Sciences, University of Michigan, Michigan, 48109, USA

## Abstract

Half-metallicity combined with wide half-metallic gap, unique ferromagnetic character and high Curie temperature has become a key driving force to develop next-generation spintronic devices. In previous studies, such half-metallicity always occurred under certain manipulation. Here, we, via examining a series of two-dimensional transition-metal trichlorides, evidenced that TiCl_3_ and VCl_3_ sheets could display exciting half-metallicity without involving any external modification. Calculated half-metallic band-gaps for TiCl_3_ and VCl_3_ sheets are about 0.60 and 1.10 eV, respectively. Magnetic coupled calculation shows that both sheets favor the ferromagnetic order with a substantial collective character. Estimated Curie temperatures can be up to 376 and 425 K for TiCl_3_ and VCl_3_ sheets, respectively. All of these results successfully disclose two new promising two-dimensional half-metallic materials toward the application of next-generation paper-like spintronic devices.

Two-dimensional (2D) nanomaterial, proposed as the versatile material, recently attracts significant interest in the scientific community^1^,^2^,^3^,^4^,^5^,^6^. Ultrathin, transparent and flexible properties render 2D nanomaterials promising and noteworthy candidates for the application of next-generation paper-like spintronic devices^7^,^8^,^9^. However, towards such an application, a key issue is to require 2D nanomaterials having the ability of completely spin-resolved electric current^10^,^11^. Half-metallicity, which has a metallic nature for one spin and a semiconducting nature for the opposite spin, can fully meet this demand^12^,^13^,^14^,^15^,^16^,^17^,^18^. As a result, a achievement of half-metallicity charmed materials scientists.

Up to now, tremendous effort has been devoted to realize the novel half-metallicity on 2D nanostructures. When graphene, BN and MoS_2_ sheets were doped by transition-metal (TM) atoms, half-metallicity could be successfully achieved^19^,^20^,^21^. It may open an effective pathway to next-generation paper-like spintronics. However, TM atoms on 2D structures are like to form the clusters, leading to the degeneration of material’s half-metallicity. To avoid this difficulty, via the introduction of zigzag edges in vacancy, Du *et al*. and Wang *et al*. successfully predicated half-metallicity in BN and MnO_2_ sheets, respectively^22^,^23^. Theoretical method seems successful in showing half-metallicity while it is still experimentally impractical because the zigzag edges of the vacancy are easy to be saturated by foreign atoms or molecules resulting the deterioration of material’s half-metallicity. The achievement of half-metallicity in h-BN and ZnO sheets by functionalizations, such as hydrogenation and fluoridation, recently also has been reported^24^,^25^, while functionalized atoms are liable to form in a random way on a host surface but not the precise arrangement leading to the decadence of material’s half-metallicity. Another effective approach to obtain the half-metallicity is to apply an external strain or voltage gate. The novel half-metallicity has been successfully predicated theoretically in strain-interacted NbS_2_ and NbSe_2_ sheets^26^, and voltage gate-interacted MnPSe_3_ sheet^27^. Nevertheless, the experimental achievement also represents a difficulty because the electronic properties of materials are extremely sensitive to the strain or voltage gate that requires a fine control of strain or voltage gate on 2D structures. Consequently, based on these analyses, an effective acquisition of half-metallic 2D materials without external conditions becomes the big challenge facing now.

Layered transition-metal trichlorides of MCl_3_ type (M = Ti, V, Cr, Fe, Mo, Ru, Rh, Ir) have been achieved for many years^28^,^29^,^30^,^31^,^32^,^33^. These crystals are stacked in an AB sequence with interlayer distances of 3.16, 3.15, 3.48, 3.17, 3.32, 3.12, 3.44, 3.30 Å for TiCl_3_, VCl_3_, CrCl_3_, FeCl_3_, MoCl_3_, RuCl_3_, RhCl_3_, IrCl_3_ structures, respectively. Relatively weak van der Waals interaction between the interlayers allows their exfoliation down to sheets by applying different technologies, as reported in graphene, phosphorene, BN sheet and MoS_2_ sheet^34^,^35^,^36^,^37^. Experimental feasibility of the exfoliation may open a new door for achieving the long-standing dream of intrinsic half-metallic sheet, since, depending on the variety of TM atoms, such MCl_3_ sheets can possess very rich electronic phases. Nevertheless, all of previous studies mainly focused on 2D carbon-group materials^38^,^39^,^40^,^41^,^42^, nitrogen-group materials^43^,^44^,^45^, transition-metal dichalcogenides^46^,^47^ and other related hybridized structures^48^,^49^,^50^,^51^, while the study for such transition-metal trichlorides was totally neglected.

In this article, two exciting two-dimensional half-metallic materials, *i.e.* TiCl_3_ and VCl_3_ sheets, were confirmed for the first time. Cleavage energy calculation shows that such 2D structures can be effectively obtained by exfoliating their bulk structures with the cleavage energy comparable to that of graphite. Interestingly, TiCl_3_ and VCl_3_ sheets can possess bellow these advanced properties: (1) the spin band-gaps for TiCl_3_ and VCl_3_ sheets can be arrived at 0.60 and 1.10 eV for TiCl_3_ and VCl_3_ sheets, respectively; (2) the induced half-metallicity for both structures presents substantial ferromagnetic character through long-rang magnetic coupling; (3) estimated Curie temperatures for TiCl_3_ and VCl_3_ sheets can be up to 376 and 425 K, respectively. These advantages render TiCl_3_ and VCl_3_ sheets with great potential for the application of next-generation paper-like spintronics.

## Results

### Exfoliated capabilities

**Figure 1:**
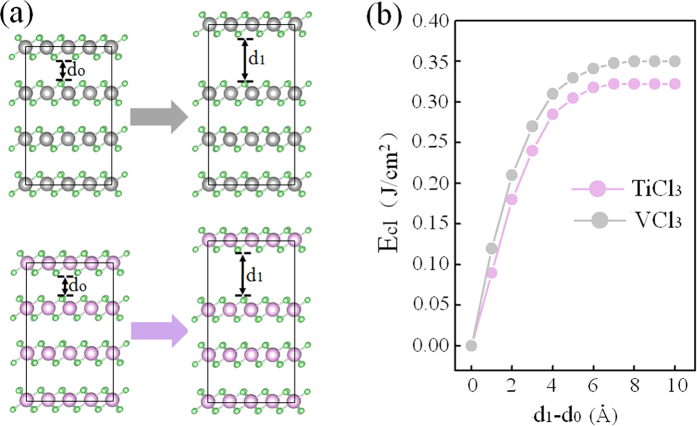
(**a**) Schematic illustration of the exfoliation procedure. (**b**) Cleavage energy, E_cl_, as a function of the separation between fractured parts.

Before studying the properties of TiCl_3_ and VCl_3_ sheets, we first tested their exfoliated capabilities. In generally, relatively small van der Waals interaction between the interlayers in TiCl_3_ and VCl_3_ sheets implies a possibility of successful exfoliations of TiCl_3_ and VCl_3_ sheets experimentally. To verity this guess, we applied an effective exfoliation method, implemented by introducing a fracture in the bulk and then calculating the corresponding exfoliated energy^52^. The reliability of this method has been widely confirmed^27^,^53^. In this method, for the exfoliation of 2D sheets from their bulk crystals, so-called cleavage decohesion energies have to be overcome^52^. As depicted in Fig. 1, such decohesion energies can be obtained by determining the asymptotic limits of exfoliated energies, which are 0.33 and 0.35 J/m^2^ for TiCl_3_ and VCl_3_ sheets, respectively. These values are quite comparable with the experimentally estimated cleavage energy of 0.36 J/m^2^ in graphite^54^, implying a possible achievement of TiCl_3_ and VCl_3_ sheets from their bulk structures experimentally. Thus, similar as for other 2D sheets, scotch tape and liquid exfoliations might be two effective methods for the production of TiCl_3_ and VCl_3_ sheets.

### Geometrical structures

**Figure 2:**
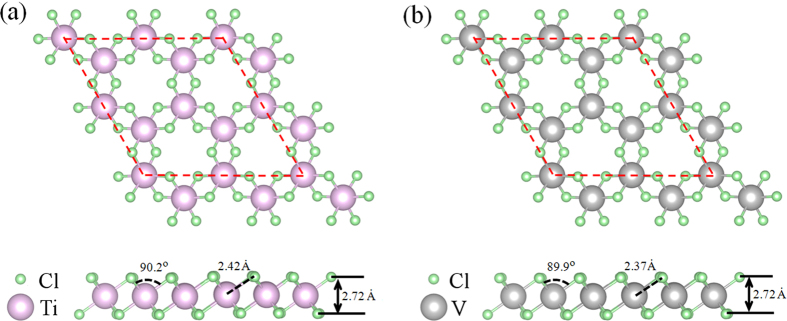
Top and side views of the optimized geometric structures of (a) TiCl_3_ and (b) VCl_3_ sheets.

We then studied the geometrical structures of TiCl_3_ and VCl_3_ sheets. Optimized geometries of TiCl_3_ and VCl_3_ sheets are shown in Fig. 2. Clearly, TiCl_3_ and VCl_3_ sheets are trilayered with a metallic atom in the middle that is covalently bonded to six Cl atoms located in the top and bottom layers forming Cl-Ti-Cl and Cl-V-Cl arrangements, respectively. Calculated lattice constant of 6.09 Å, Cl-Ti bond length of 2.42 Å, Cl-Cl distance of 2.72 Å and Cl-Ti-Cl angle of 90.2° in TiCl_3_ sheet are quite comparable with those found in TiCl_3_ crystal^28^, and calculated lattice constant of 6.01 Å, Cl-V bond length of 2.37 Å, Cl-Cl distance of 2.72 Å and Cl-V-Cl angle of 89.9° in VCl_3_ sheet are quite comparable with those found in VCl_3_ crystal^29^. As a result, TiCl_3_ and VCl_3_ sheets remain the geometries as those found in TiCl_3_ and VCl_3_ crystals very well, implying an structural rigidity of such sheets after exfoliation.

### Half-metallicity

**Figure 3:**
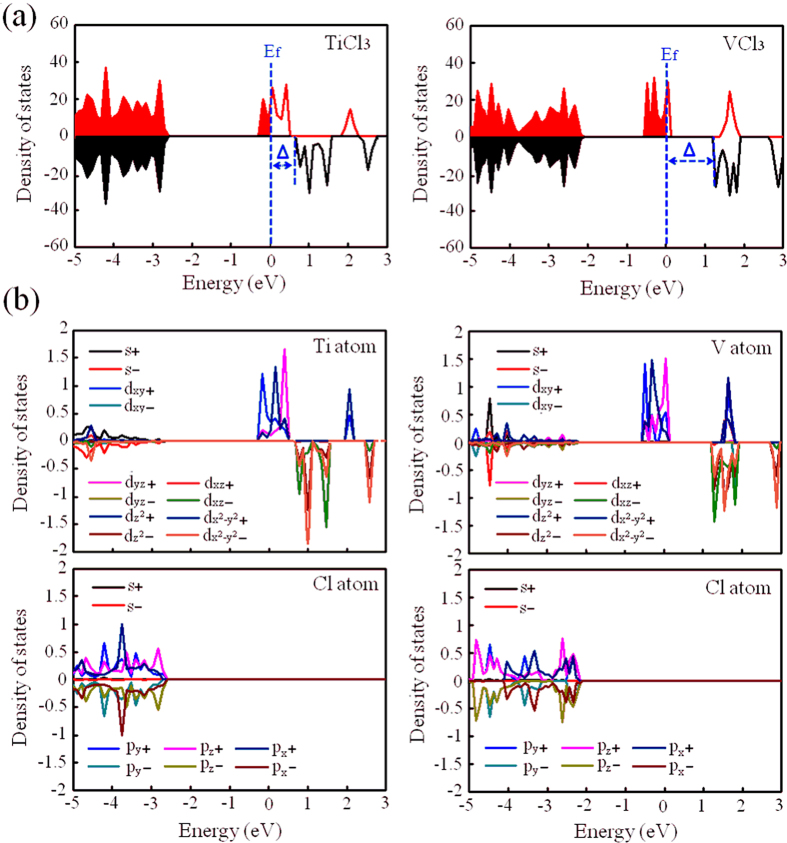
(**a**) Spin-polarized total density of states of TiCl_3_ and VCl_3_ sheets. (**b**) Spin-polarized projected density of states of Cl and TM atoms in TiCl_3_ and VCl_3_ sheets.

In order to explore if TiCl_3_ and VCl_3_ sheets can possess the half-metallic character, we plotted their spin-polarized total density of states (TDOS), as listed in Fig. 3a. Luckily, the novel half-metallicity was observed for both structures. The spin-up electrons are metallic around the Fermi level while the spin-down electrons are semiconducting. Such a polarized character opens a transport channel for spin-up electrons and blocks the channel for spin-down electrons, ensuring a 100% passage of preferred spin. Note that, comparing with previous studies where half-metallicity occurred under certain external constraints, the half-metallicity found here is totally intrinsic without any external constraints, meaning that TiCl_3_ and VCl_3_ sheets should be more suitable for actual spin applications. In order to understand the half-metallic character in more detail, we investigated the atomic projected density of states (PDOS), as listed in Fig. 3b. As demonstrated by the states of entire energy regions for TM atom and Cl atom in both sheets, the states of TM atom and Cl atom can be divided into two parts: one part related with the energies from −2.0 to −5.0 eV (identified as part 1) and the other part related with the energies around the Fermi level (identified as part 2). For part 1, a strong hybridization was found in both sheets, mainly contributed by Ti’s s, d_xz_, d_x2−y2_ states and Cl’s p_x_, p_y_ and p_z_ states. It has been revealed in our previous studies that strong hybridized interaction can certainly reduce or quench magnetism of TM atom^26^. Thus, these states don’t contribute spin-polarization, in which spin-up states match spin-down states very well. For part 2, the situation is quite different. Because of the deficiency of states at Cl atom, states of TM atom in both sheets, contributed by Ti’s d_xy_, d_yz_, d_z2_ states, are not hybridized. Consequently, these states present a spin-polarization, in which spin-up states don’t match spin-down states. Based on these results, we can conclude that half-metallicity in TiCl_3_ and VCl_3_ sheets mainly comes from the non-hybridized TM’s 3*d* states while the contribution from hybridized TM’s 3*d* states can be neglected.

### Half-metallic gap

To achieve the great promise for the use of half-metallicity in spintronic devices, wide half-metallic gap is extremely important^55^,^56^. Thus, we valuated the values of half-metallic gap for TiCl_3_ and VCl_3_ sheets. Calculated half-metallic gaps, labeled as Δ in Fig. 3a, are about 0.60 and 1.10 eV for TiCl_3_ and VCl_3_ sheets, respectively. In order to conveniently compare with previous works, we also used density functional theory (DFT) method to estimate the half-metallic gap. Values, employed by DFT, for TiCl_3_ and VCl_3_ sheets are about 0.42 and 0.64 eV, respectively, which are comparable with the values of 0.46 eV in half-metallic LaMn_0.5_Zn_0.5_AsO_0.5_H_0.5_ alloy and 0.50 eV in half-metallic Sr_2_FeMoO_6_ and Sr_2_FeReO_6_ alloys^57^,^58^,^59^. Note that, the bandgaps are also dependent on the choice of parameters. Thus, wide half-metallic gaps here seems survived to the choice of functional, which provides a great capability for the prevention of spin flip transition, a tremendous challenge, that might occur at a low temperature.

### Ferromagnetic coupling

**Figure 4:**
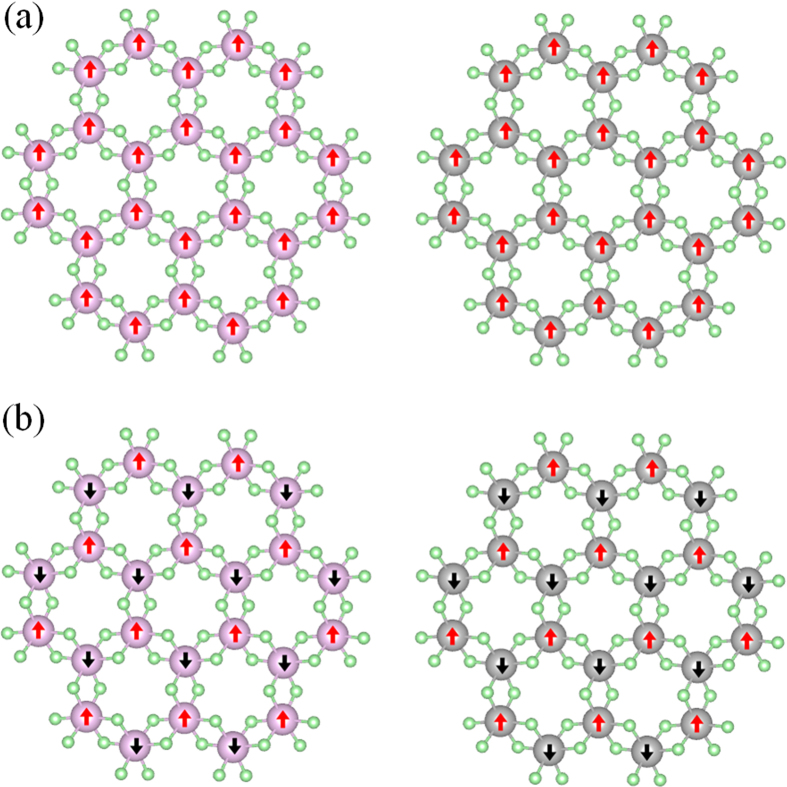
Schematic illustrations of (**a**) ferromagnetic and (**b**) antiferromagnetic couplings in TiCl_3_ and VCl_3_ sheets.

**Figure 5:**
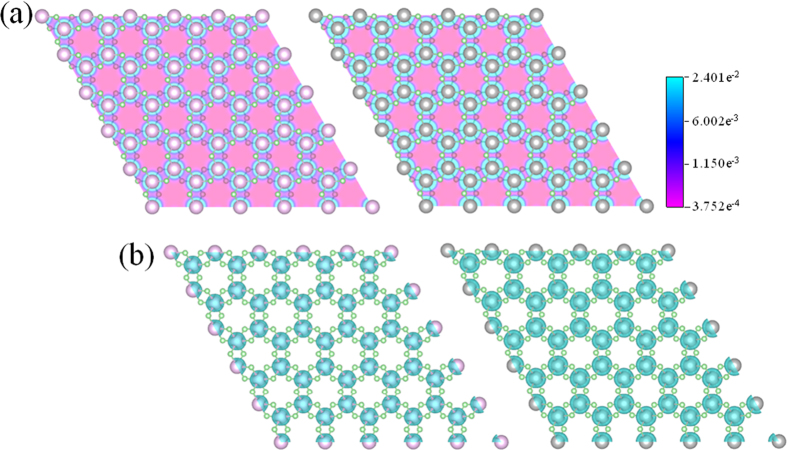
Spin densities of (**a**) two-dimensional plot and (**b**) isosurface in TiCl_3_ and VCl_3_ sheets (isovalue 0.02 e/Å^3^).

Ferromagnetic coupling is another vital property for the application of half-metallic materials in spintronic devices^60^,^61^,^62^. Bearing this in mind, we considered two different coupling configurations, namely, ferromagnetic (FM) coupling and antiferromagnetic (AFM) coupling, as listed in Fig. 4. After optimization, we found that the energy of FM state in TiCl_3_ sheet lies 780 eV lower than that of AFM state, and the energy of FM state in VCl_3_ sheet lies 880 eV lower than that of AFM state. Since our calculations are based on a supercell that consists of four unit cells, the energy of FM state for one unit cell composed of two TM atoms and six Cl atoms is lower than that of AFM state by 195 and 220 meV for TiCl_3_ and VCl_3_ sheets, respectively, clearly showing that the FM coupling is favored for both sheets. In order to understand the strength of ferromagnetism with the distance, we calculated strain-dependent energy difference between AFM coupling and FM coupling in Figure 1S. It shows that the difference of energy between AFM coupling and FM coupling increases with the tensile strain for both cases. This finding is in agreement with the result found in strain-interacted VS_2_ and VSe_2_ sheets and can be attributed to the competitive effect between the change of through-bond interaction and the change of through-space interaction with the tensile strain^63^. To visualize the spin distribution of FM coupling on TiCl_3_ and VCl_3_ sheets, we plot their spin densities, *i.e.* the charge density difference between spin-up and spin-down channels, as listed in Fig. 5. Agreeing with the magnetic moment analysis, the induced spin polarization is mainly contributed by TM atoms while the contribution from Cl atoms can be neglected for both sheets. Note that, the unpaired spin electrons in TiCl_3_ and VCl_3_ sheets exhibit a substantial collective character, which is crucially important for the applications of system in spintronic devices but is often overlooked in the previous studies. Thus, the finding of unique FM coupling here might render TiCl_3_ and VCl_3_ sheets more practical applications to spintronic devices.

### Curie temperature

**Figure 6:**
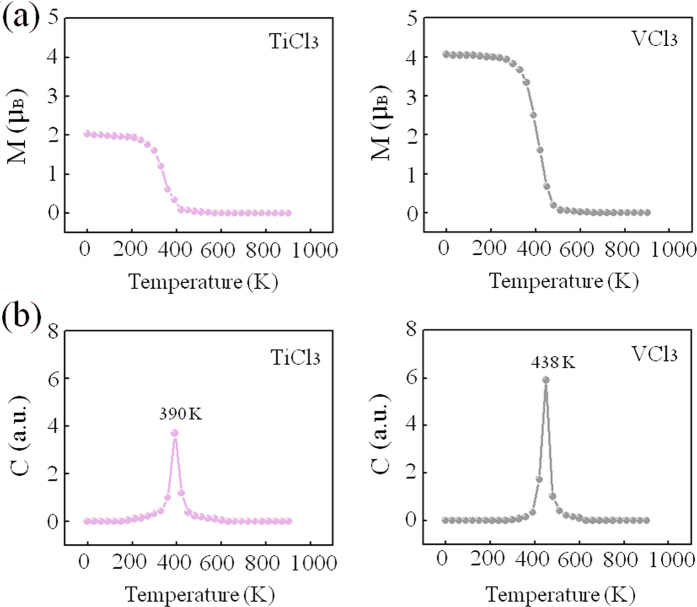
Variations of (**a**) the magnetic moment per cell, M, and (**b**) heat capacity, C, as a function of temperature in TiCl_3_ and VCl_3_ sheets.

Considering the practical application of half-metallicity in spintronic devices, Curie temperature of materials is the other important property that should be comparable to or higher than room temperature^64^. To this end, we finally estimate the Curie temperature T_C_, based on the Heisenberg model with the expression of k_B_T_C_ = (2/3)ΔE, where k_B_ is the bolzmann constant, Tc is the Curie temperature and ΔE is the energy difference between FM state and AFM state^65^. According to this model, we found the values of 376 and 425 K for TiCl_3_ and VCl_3_ sheets, respectively. Obviously, half-metallic TiCl_3_ and VCl_3_ sheets can be utilized at room temperature. In order to directly verify such an exciting result, Monte Carlo simulation was also carried out. Before the Monte Carlo simulation, the exchange coupling constant, J, was firstly considered according to the Ising theory, H = −J∑m_i_m_j_, where m_i_ and m_j_ are the magnetic moments at sites i and j, and H is the Hamiltoninan. For TiCl_3_ and VCl_3_ structures here, this formula can be written as J = ΔE/64m^2^, where ΔE is the energy difference between FM state and AFM state and m = |m|. Calculated exchange coupling constants are about 12.2 and 3.4 meV for TiCl_3_ and VCl_3_ sheets, respectively. Then the Monte Carlo simulation was lasted for 5 × 10^5^ loops with a 100 × 100 supercell. Temperature-dependent magnetic moment curve and heat capacity curve are listed in Fig. 6a,b, respectively. As shown in Fig. 6a, Curie temperature of TiCl_3_ sheet can be evaluated to the value between 300 K at which the magnetic moment of TiCl_3_ sheet starts dropping gradually and 400 K at which the paramagnetic state of TiCl_3_ sheet is achieved, and Curie temperatures of VCl_3_ sheet can be evaluated to the value between 350 K at which the magnetic moment of VCl_3_ sheet starts dropping gradually and 450 K at which the paramagnetic state of VCl_3_ sheet is achieved. As shown in Fig. 6b, via locating the peak position of the heat capacity curve, Curie temperatures of TiCl_3_ and VCl_3_ sheets were precisely determined to be 390 and 438 K, respectively. In generally, Monte Carlo simulation is easy to give a Curie temperature of 0 K for 2D system. However, considering that the interlayer magnetic couplings through the van der Waals gap in bulk TiCl_3_ and VCl_3_ crystals are vanishingly small, the Curie temperatures of bulk TiCl_3_ and VCl_3_ crystals are dominated by nearest-neighbor exchange interactions. Thus, Curie temperatures of TiCl_3_ and VCl_3_ sheets seem equal to those of bulk TiCl_3_ and VCl_3_ crystals. This may explain why TiCl_3_ and VCl_3_ sheets can present certain Curie temperatures. As a result, Heisenberg model and Monte Carlo simulation give the similar results, confirming the validity of our estimated Curie temperature. Based on the result of such high curie temperatures found in TiCl_3_ and VCl_3_ sheets, explaining the origin of this property is important. According to the Heisenberg model, ΔE, the energy difference between FM state and AFM state, plays a key role for the magnitude of Curie temperature. Previous studies have shown that the FM state of TM atoms in such system was caused by the through-bond coupling interaction via which an atom with spin-up (spin-down) density would induce a spin-down (spin-up) density on the adjacent atom bonded to it by the expression of “…M_TM_ → -M_Cl_ → M_TM_…” and the AFM coupling of TM atoms in such system was caused by the through-space coupling via which an atom with spin-up (spin-down) density would induce a spin-down (spin-up) density on the nearest-neighboring atom directly without mediation atom by the expression of “…M_TM_ → -M_TM_…”^63^. Here M_TM_ and M_Cl_ denote the magnetic moment of TM and Cl atoms, respectively. Thus, based on such an analysis, the value of ΔE in both structures can be recognized as a competitive effect of through-bond coupling interaction and through-space coupling interaction. When the difference between through-bond interaction and through-space interaction is distinct, the value of ΔE is large, otherwise small. Note that, for both structures, spin electrons contributed by TM’s 3*d* states are rather localized which leads to a very slight through-space coupling interaction while the bond between TM and Cl atoms is rather strong which induces a very strong through-bond coupling interaction. Naturally, distinct difference between through-bond coupling interaction and through-space coupling interaction gives a large value of ΔE, resulting in the high Curie temperature.

## Discussion

In this work, we, via examining a series of transition-metal trichlorides, identified two intrinsic two-dimensional half-metallic materials: TiCl_3_ and VCl_3_ sheets. Cleavage energy calculation shown such 2D structures can be effectively obtained by exfoliating their bulk structures. In contrast to previous studies, the half-metallicity found in TiCl_3_ and VCl_3_ structures has the following advantages: (1) it is not necessary to substitutionally or adsorptionly dope 2D structure by TM atoms; (2) it is not necessary to induce zigzag edges in 2D structure by fabricating vacancy; (3) it is not necessary to functionalize the 2D structure, such as hydrogenation and fluoridation; (4) it is not necessary to apply an external strain or voltage gate to the 2D structure. Although all these methods can make 2D structures successfully obtain half-metallicity, experimental effective manipulations are still difficult. Further studies show that (1) the spin band-gaps for TiCl_3_ and VCl_3_ sheets can be arrived at 0.60 and 1.10 eV for TiCl_3_ and VCl_3_ sheets, respectively; (2) the induced half-metallicity for both structures favors ferromagnetic coupling; (3) the estimated Curie temperatures for TiCl_3_ and VCl_3_ sheets can be up to 376 and 425 K, respectively. Therefore, based on these analyses, the intrinsic half-metallicity combined with wide half-metallic gap, unique ferromagnetic character and high Curie temperature render TiCl_3_ and VCl_3_ sheets with great potential for the application of next-generation paper-like spintronics.

## Methods

### DFT calculations

The first-principle calculations are performed using the Vienna *ab initio* simulation package (VASP). Note that, in order to count the electron correlation effects of Ti’s and V’s 3*d* orbitals and obtain precise electronic and magnetic properties of TiCl_3_ and VCl_3_ sheets, we employ the screened hybrid HSE06 functional, which includes the accurate Fock exchange and thus performs much better than the DFT method. In our calculations, a 2 × 2 supercell was used for both TiCl_3_ and VCl_3_ structures. The electronic wave functions were expanded using a plane-wave basis set with a cutoff energy of 500 eV. The pseudopotentials with 3d^2^4s^2^, 3d^3^4s^2^ and 3s^2^3p^5^ valence electron configurations were used for Ti, V and Cl atoms, respectively. For bulk TiCl_3_ and VCl_3_ crystals, van der Waals interaction was considered, and the corresponding brillouin zone integration was performed with a 6 × 6 × 2 *k*-point grid. For TiCl_3_ and VCl_3_ sheets, a vacuum space of 15 Å was used to avoid the interaction between images, and the corresponding brillouin zone integration was performed with a 10 × 10 × 1 *k*-point grid. All the calculations were carried out with spin-polarization. The atomic positions and lattice constants of the structures were relaxed until all the force components were smaller than 0.01 eV/Å. Here the spin-orbital coupling correcting was not considered, since our test shown that it had little influence on our results.

## Additional Information

**How to cite this article**: Zhou, Y. *et al*. Evidencing the existence of exciting half-metallicity in two-dimensional TiCl_3_ and VCl_3_ sheets. *Sci. Rep.* **6**, 19407; doi: 10.1038/srep19407 (2016).

## Supplementary Material

Supplementary Information
