# Supplementary material for: Evidencing the existence of exciting half-metallicity in two-dimensional TiCl3 and VCl3 sheets
Source: Sci Rep. 2016 Jan 18;6:19407. doi: 10.1038/srep19407 (PMC4726018; doi:10.1038/srep19407)
Supplement: Supplementary Information [file srep19407-s1.doc]

**Supplementary information**

**“Evidencing the existence of exciting half-metallicity in two-dimensional TiCl_3_ and VCl_3_ sheets”** by Yungang Zhou, Haifeng Lu, Xiaotao Zu, & Fei Gao


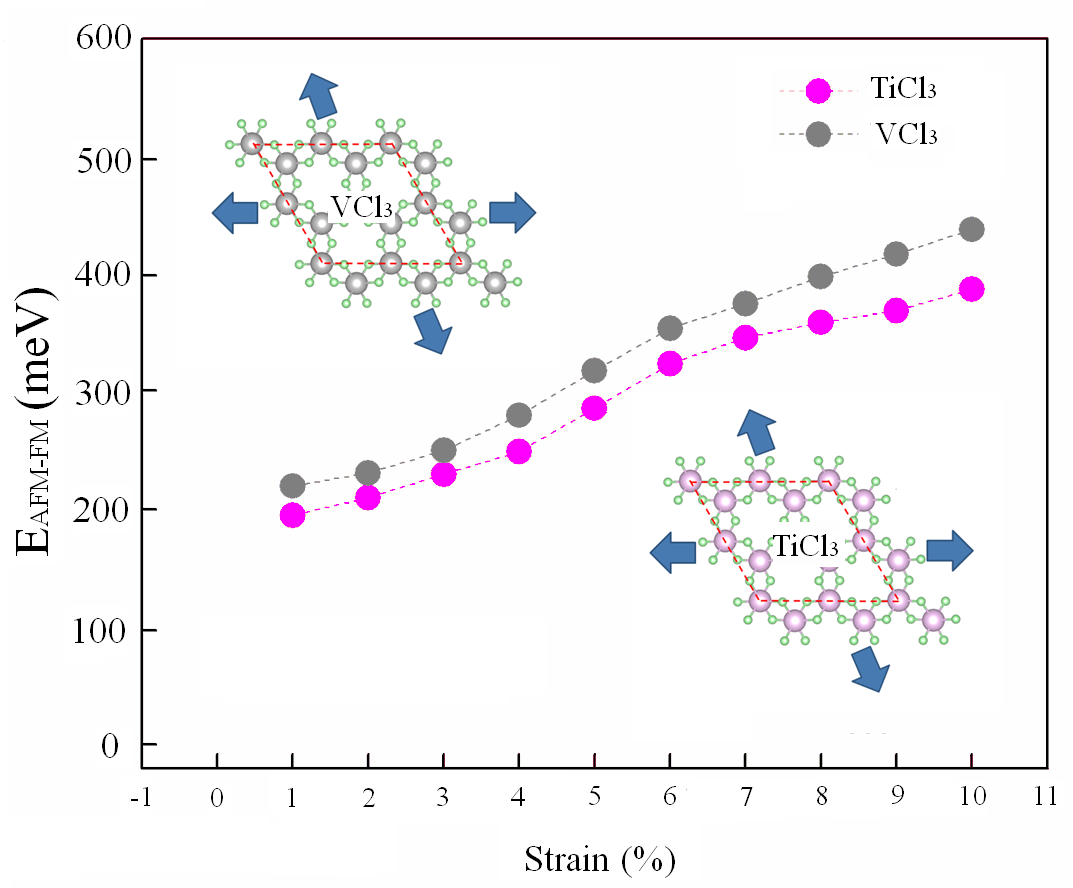


Figure 1S. Strain dependence of the energy difference per unit cell between AFM coupling and FM coupling for TiCl_3_ and VCl_3_ structures.
